# The South African Hospitals Commission

**Published:** 1901-02-02

**Authors:** 


### The Hospital

A Journal of

The Medical Sciences and Hospital Administration.

Vol. XXIX.—No. 749.] Edited by Sir Henry Burdett, K.C.B., and Solomon C. Smith, M.D., M.R.C.P. [February 2, 1901.

### The South African Hospitals Commission.

Mr. Burdett-Coutts protests against the findings of the Hospitals Commission, saying that both the report and the inquiry upon which it has been based are open to the gravest objections. We think, however, that, putting personal matters entirely on one side, he has good reason to be satisfied with the part he has played in causing this investigation to be undertaken; in bringing to light a condition of affairs which we are certain that the great mass of the British public was entirely unprepared for; and in forcing on an inquiry which is sure to have far-reaching effects in reforming the weak spots in the organisation of the Medical Department.

The report is too large a document for us to attempt an analysis of its contents at this moment, especially as the evidence on which it is based is not yet printed; but we rise from its perusal with a strong feeling that all was not done for the sick and wounded in South Africa last year that might have been done under a better system. Although to a certain extent, and up to a certain point, the excuse of lack of transport was a sufficient one, and although it is true that war must always have its dark side, still, in reading between the lines of a report which on every page shows evidence of an unwillingness to use hard words, we cannot but see that much suffering was due on the one hand to the failure to send up surgeons, nurses, and medical stores which might have been sent up, and on the other hand to the lack of initiative on the part of some of the medical authorities, especially at Bloemfontein, in not purchasing and commandeering the things which they were short of, and, more strange still, in not obtaining—as it is evident they might have obtained—many things which were actually available if they had been asked for.

About one hospital in particular (No. 8) especial complaints were made. There undoubtedly was good excuse for much discomfort, for it was desperately overcrowded. Still it is clear that much more might have been done than was done for the comfort of the patients. We are told that the supply of nurses was "very insufficient," that "men were not properly nursed," and that "typhoid patients, though probably not of a severe type, had in some instances to wash and tend themselves," &c. Is it not then a surprising thing to find Lieutenant-Colonel Beamish, the principal medical officer of this hospital, saying in his evidence that "he, generally speaking, got all the nurses he asked for"? Why on earth, then, did he not ask for more?

Take, then, the question of blankets, of which "undoubtedly there was a shortage" for a time. Yet "there was at all times in this hospital a sufficient store of blankets, and beyond all question there was a sufficient supply in the principal headquarters' stores in Bloemfontein." The Commissioners say that "those who noticed the deficiency do not appear to have known to whom to apply to get the blankets that *were in store*." Was there nobody among those in command with sufficient energy to run around and get those blankets which actually were in store?

Nor can we admit that this *laisser faire* system was confined to the principal medical officers of this unfortunate hospital. It was well known that there was great friction between some of the civil surgeons in this hospital and the officers of the Royal Army Medical Corps, "and even between the senior officers of the Royal Army Medical Corps themselves;" yet nothing seems to have been done to put a stop to a condition of affairs which was especially scandalous in view of the terrible surroundings in the midst of which these squabbles were allowed to continue. Nothing could better show the evils of the seniority system which has been allowed to take possession of the Army Medical Department than the fact that this friction was known and yet was allowed to go on. Surgeon-General Stevenson, the principal medical officer, admitted that he knew of it, and "believed that the hospital would get into difficulties in consequence of it," and, indeed, said that he thought that "the friction would go on until the machine stopped." Colonel Exham also, who succeeded Surgeon-General Stevenson, "knew of the hostile feelings which were entertained by the different members of the Staff towards each other," and yet nothing effectual was done. "To the Commission it is a matter for surprise that more vigorous steps were not taken by these officers to put an end to a condition of affairs which could only result in disorganisation," and their surprise will be shared by every sensible man.

Passing now from what one may call the historical portions of the report to the recommendations made with a view of remedying the defects discovered, and reading them, as we fairly may do, as indicating the existence of corresponding deficiencies in the system at present in operation, we see an admission of almost everything that has been said against the existing arrangements: (1) the undermanning of the whole service, so that its numbers and its equipment are not sufficient even for ordinary duties in time of peace; (2) the lack of all arrangements by which medical officers and orderlies can be added to the regular staff in case of a great war; (3) the drawbacks which make the service fail to attract a sufficient and regular supply of officers of good professional attainments; (4) the ill-treatment of the service in regard to the holiday leave granted to its officers; (5) the evils arising from the absence of proper provision for enabling its officers to "become adequately acquainted with the advancements in medical and surgical science"; (6) the danger arising from promotion by seniority in the higher ranks; (7) the want of trained female nursing in the fixed hospitals; and (8) the lack of specially trained sanitary officers. Many other things are suggested, and although the Commissioners end their report with a carefully worded eulogy, one cannot fail to see that the numerous recommendations made really mean how numerous were the defects which were discovered in the system, and in the relation of the medical to the other departments in the Army.

